# Correction: The Theory of Planned Behavior during the COVID-19 pandemic: A comparison of health behaviors between Belgian and French residents

**DOI:** 10.1371/journal.pone.0315839

**Published:** 2024-12-11

**Authors:** Robin Wollast, Mathias Schmitz, Alix Bigot, Olivier Luminet

The following information is missing from the Funding statement: This research has been financially supported by the Louvain Foundation.

An additional affiliation is missing for the fourth author. Olivier Luminet is also affiliated with the Belgian Fund for Scientific Research (FRS-FNRS).

The ORCID iDs are missing for the second, third and fourth author. Please see the authors’ respective ORCID iDs here:

- Author Mathias Schmitz’s ORCID iD is: 0000-0001-9272-5874

(https://orcid.org/0000-0001-9272-5874).

- Author Alix Bigot’s ORCID iD is: 0000-0002-7841-7653

(https://orcid.org/0000-0002-7841-7653).

- Author Olivier Luminet’s ORCID iD is: 0000-0002-1519-2178

(https://orcid.org/0000-0002-1519-2178).
